# Draft Genome Sequences of *Stenotrophomonas maltophilia* Strains Sm32COP, Sm41DVV, Sm46PAILV, SmF3, SmF22, SmSOFb1, and SmCVFa1, Isolated from Different Manures in France

**DOI:** 10.1128/genomeA.00841-16

**Published:** 2016-08-18

**Authors:** Josselin Bodilis, Benjamin Youenou, Jérome Briolay, Elisabeth Brothier, Sabine Favre-Bonté, Sylvie Nazaret

**Affiliations:** a Research Group on Environmental Multi-Resistance and Efflux Pump, CNRS, École Nationale Vétérinaire de Lyon, and Université Lyon 1 UMR 5557 Ecologie Microbienne, Villeurbanne, France; b Plateforme du DTAMB, CNRS, Université Lyon 1, Villeurbanne, France

## Abstract

*Stenotrophomonas maltophilia* is a major opportunistic human pathogen responsible for nosocomial infections. Here, we report the draft genome sequences of Sm32COP, Sm41DVV, Sm46PAILV, SmF3, SmF22, SmSOFb1, and SmCVFa1, isolated from different manures in France, which provide insights into the genetic determinism of intrinsic or acquired antibiotic resistance in this species.

## GENOME ANNOUNCEMENT

*Stenotrophomonas maltophilia* is a ubiquitous bacterium that can be found in various environments (1). However, *S. maltophilia* is also an opportunistic pathogen responsible for numerous nosocomial infections, and it exhibits high resistance levels toward most of the currently used antimicrobial agents (2). Although environmental *S. maltophilia* usually presents lower levels of resistance to antibiotics than clinical strains, multidrug-resistant (MDR) isolates have been isolated from soils and aqueous environments (3–5). Like most other bacterial pathogens, the major intrinsic resistance mechanism responsible for its MDR phenotype can be attributed to the activity of chromosomally encoded multidrug efflux pumps (5–7).

Sm32COP, Sm41DVV, and Sm46PAILV were isolated from the compost of horse manure, and SmF3 and SmF22 were isolated from cattle manure in Feucherolles. SmSOFb1 and SmCVFa1 were isolated from horse manure in Saint Olive and cattle manure in Versailleux, respectively. Six of the seven strains harbor the MDR phenotype (4). Only Sm32COP is susceptible to most of the antibiotics tested (Table 1).

**TABLE 1:** Summary information for the draft genome sequences of seven *Stenotrophomonas maltophilia* strains from different types of manure collected in various farms in France

Strain	Source of isolation	Geographic origin	Antibiotic phenotype	Genome size (bp)	G+C content (%)	No. of contigs	Accession no.
Sm32COP	Compost of horse manure	Feucherolles	Susceptible	4,548,960	66.4	45	LYVH00000000
Sm41DVV	Compost of horse manure	Feucherolles	MDR	4,139,723	66.9	26	LYVI00000000
Sm46PAILV	Compost of horse manure	Feucherolles	MDR	4,123,397	66.7	51	LYVJ00000000
SmF3	Cattle manure	Feucherolles	MDR	4,595,297	66.5	77	LYVK00000000
SmF22	Cattle manure	Feucherolles	MDR	4,583,062	66.4	64	LYVL00000000
SmSOFb1	Horse manure	Saint Olive	MDR	4,483,386	66.4	93	LZPC00000000
SmCVFa1	Cattle manure	Versailleux	MDR	4,264,176	66.8	30	LZPD00000000

Genome sequencing for the seven strains was performed using an Illumina MiSeq PE 2 × 300 platform at the University of Lyon (France). For each strain, between 2,827,464 and 3,703,116 paired-end reads with a mean length of about 250 bp after trimming were obtained, and coverage between 166× and 222× was generated (Table 1). Reads were *de novo* assembled using five different genome assemblers (SPAdes, Celera, Minia, Velvet, and MaSuRCA), except for SmF3 and SmF22. Contigs from these assemblers were merged using CISA (8). For SmF3 and SmF22, reads were assembled using only the SPAdes software version 3 (9).

The draft genomes of Sm32COP, Sm41DVV, Sm46PAILV, SmF3, SmF22, SmSOFb1, and SmCVFa1 have total sizes ranging from 4,123,397 to 4,583,062 bp, with a G+C content between 66.4% and 66.9%, and consist of 26 to 93 contigs with a size greater than 200 bp (Table 1). The contigs were analyzed using the NCBI Prokaryotic Genome Annotation Pipeline (PGAP) (http://www.ncbi.nlm.nih.gov/genome/annotation_prok). In total, between 3,580 and 4,068 predicted protein-coding sequences (CDSs) and between 68 and 75 tRNA genes were found among the seven genomes. These general features are consistent with those observed in other *S. maltophilia* strains (5).

We previously studied the antibiotic resistance gene content in *S. maltophilia* strains of various origins, with a special emphasis on resistance-nodulation-division (RND) efflux pumps (5). The new genomes described here encode eight or nine RND pumps putatively involved in MDR, with seven RND pumps conserved in all *S. maltophilia* strains. Interestingly, the SmeABC MDR pump is absent in three strains, unrelated to their antibiotic resistance phenotype, while some additional strain-specific RND efflux pumps were detected. Functional redundancies and/or specific regulation explain likely the variation of resistance phenotype observed in these strains and should be further studied.

### Accession number(s).

These draft genome sequences have been deposited at DDBJ/GenBank/EMBL under accession numbers listed in Table 1. The versions described in this paper are the first versions.

## References

[1] Brooke JS 2012 *Stenotrophomonas maltophilia*: an emerging global opportunistic pathogen. Clin Microbiol Rev 25:2–41. doi:10.1128/CMR.00019-11. 22232370 PMC3255966

[2] Sánchez MB 2015 Antibiotic resistance in the opportunistic pathogen *Stenotrophomonas maltophilia*. Front Microbiol 6:658. doi:10.3389/fmicb.2015.00658. 26175724 PMC4485184

[3] Berg G, Roskot N, Smalla K 1999 Genotypic and phenotypic relationships between clinical and environmental isolates of *Stenotrophomonas maltophilia*. J Clin Microbiol 37:3594–3600. 10523559 10.1128/jcm.37.11.3594-3600.1999 PMC85701

[4] Deredjian A, Alliot N, Blanchard L, Brothier E, Anane M, Cambier P, Jolivet C, Khelil MN, Nazaret S, Saby N, Thioulouse J, Favre-Bonté S 2016 Occurrence of *Stenotrophomonas maltophilia* in agricultural soils and antibiotic resistance properties. Res Microbiol 167:313–324. doi:10.1016/j.resmic.2016.01.001. 26774914

[5] Youenou B, Favre-Bonté S, Bodilis J, Brothier E, Dubost A, Muller D, Nazaret S 2015 Comparative genomics of environmental and clinical *Stenotrophomonas maltophilia* strains with different antibiotic resistance profiles. Genome Biol Evol 7:2484–2505. doi:10.1093/gbe/evv161. 26276674 PMC4607518

[6] Zhang L, Li XZ, Poole K 2000 Multiple antibiotic resistance in *Stenotrophomonas maltophilia*: involvement of a multidrug efflux system. Antimicrob Agents Chemother 44:287–293. doi:10.1128/AAC.44.2.287-293.2000. 10639352 PMC89673

[7] Blair JM, Piddock LJ 2009 Structure, function and inhibition of RND efflux pumps in gram-negative bacteria: an update. Curr Opin Microbiol 12:512–519. doi:10.1016/j.mib.2009.07.003. 19664953

[8] Lin SH, Liao YC 2013 CISA: contig integrator for sequence assembly of bacterial genomes. PLoS One 8:e60843. doi:10.1371/journal.pone.0060843. 23556006 PMC3610655

[9] Bankevich A, Nurk S, Antipov D, Gurevich AA, Dvorkin M, Kulikov AS, Lesin VM, Nikolenko SI, Pham S, Prjibelski AD, Pyshkin AV, Sirotkin AV, Vyahhi N, Tesler G, Alekseyev MA, Pevzner PA 2012 SPAdes: a new genome assembly algorithm and its applications to single- cell sequencing. J Comput Biol 19:455–477. doi:10.1089/cmb.2012.0021. 22506599 PMC3342519

